# Aggressive Serous Carcinomas of the Female Reproductive Tract: Cancer-Prone Cell States and Genetic Drivers

**DOI:** 10.3390/cancers17040604

**Published:** 2025-02-11

**Authors:** Daryl J. Phuong, Matalin G. Pirtz, Coulter Q. Ralston, Benjamin D. Cosgrove, John C. Schimenti, Andrea Flesken-Nikitin, Alexander Yu. Nikitin

**Affiliations:** 1 Department of Biomedical Sciences, Cornell University, Ithaca, NY 14853, USA; djp299@cornell.edu (D.J.P.); mgp73@cornell.edu (M.G.P.); cqr3@cornell.edu (C.Q.R.); jcs92@cornell.edu (J.C.S.); 2 Department of Molecular Biology and Genetics, Cornell University, Ithaca, NY 14853, USA; 3 Meinig School of Biomedical Engineering, Cornell University, Ithaca, NY 14853, USA; bdc68@cornell.edu

**Keywords:** cancer-prone cell state, genetic drivers, ovarian cancer, uterine cancer

## Abstract

In 2025, gynecological cancers are projected to account for approximately 10% of cancer-related deaths in women. High-grade serous ovarian carcinoma (HGSC) and serous endometrial carcinoma (SEC) are the most lethal gynecological cancer subtypes. Both malignancies commonly have *TP53* mutations, alterations of the RB1 pathway, and numerous secondary mutations. Both carcinoma types consist of poorly differentiated and highly heterogeneous cell populations at the time of detection. Latent development and rapid progression of HGSC and SEC impede the identification of definitive cells of origin and genetic drivers. Here, we review our current knowledge about cancer-prone cell states and genetic drivers. We also discuss how emerging transcriptomic and genetic tools applied to contemporary model systems may facilitate the identification of novel targets for timely detection and therapeutic intervention.

## Simple Summary

Gynecological cancers significantly contribute to cancer-related deaths in women, with serous carcinomas being the primary subtypes responsible for high mortality. Here we review the most aggressive female reproductive cancers - high-grade serous ovarian carcinoma and serous endometrial carcinoma. We examine advancements in identifying cancer-prone cell states in the ovary, uterine tube, and uterus, along with the genetic factors that drive malignant transformation. A comparative analysis of these diseases highlights both shared and distinct mechanisms influencing cancer formation. Finally, we discuss innovative research techniques that may enhance our understanding of these malignancies. By elucidating the role of cancer-prone cell states and genetic drivers in disease onset, we can uncover new therapeutic targets and strategies to improve patient outcomes.

## 1. Introduction

Cancers of the ovary and uterine corpus contribute to approximately 10% of cancer-related deaths in women today [1]. High-grade serous ovarian carcinoma (HGSC) and serous endometrial carcinoma (SEC) are among the most aggressive forms of cancer affecting women. They currently account for 80% of ovarian and 40% of uterine cancer-related deaths, respectively [2,3]. HGSC is the most aggressive and prevalent subtype of ovarian cancer. Consequently, HGSC is most often detected in advanced stages with a 5-year survival rate of 32%; however, patients diagnosed during early stages of HGSC have a 5-year survival rate of 71% [4]. Furthermore, HGSC contributes to a significant proportion of cancer-related deaths despite a relatively low incidence rate affecting only 1.3% of women [4]. SEC is slightly more common than HGSC, as uterine cancers are the fourth most common cancer among women in the US, and about 10% of those cases are diagnosed as SEC [1,2]. Similarly to HGSC, the overall 5-year survival rate for SEC is about 30–40% but significantly improves when identified early to as high as 80% [5]. Furthermore, for poorly understood reasons, the mortality rate has increased in SEC patients over past decade [6,7].

Improved understanding of the key molecular and cellular mechanisms involved in the initiation and progression of these malignancies offers promise for the identification of new diagnostic markers and targets for early treatment and prevention. Unfortunately, HGSC progresses latently and commonly remains undetected until advanced stages of neoplastic progression, after the disease has spread. Although SEC can be detected earlier than HGSC due to abnormal bleeding, 30–50% of cases are nevertheless diagnosed at stage III or IV, specifically, when disease has spread to areas outside of the uterus [8,9,10,11,12,13]. At detection, these malignancies consist of poorly differentiated and highly heterogeneous cell populations. Such cells are insufficiently reminiscent of their cell of origin and contain numerous mutations of unproven importance for carcinogenesis. As a result, advancements in early detection and intervention methods have been hindered. 

HGSC and SEC share common morphological features, such as high nuclear to cytoplasmic (N/C) ratio, conspicuous nuclear atypia, high mitotic index, complex papillary or glandular architecture, slit-like spaces, and hobnail cells [14,15]. During recent years some progress has been made in the identification of putative precursor lesions of HGSC and SEC. However, further cell fate studies are required to definitively link these lesions to overt carcinomas. Furthermore, modeling of early cancer stages is complicated by our insufficient knowledge about normal cell lineages of target tissues and genetic cancer drivers critical for the transformation of individual states of cell differentiation. 

In this review, we summarize the current knowledge surrounding cancer-prone cell states and genetic drivers. We also discuss how new interdisciplinary approaches may improve our comparative understanding of early mechanisms of HGSC and SEC formation, thereby unraveling new prognostic and diagnostic tools and therapeutic modalities. 

## 2. Cancer-Prone Cell States

### 2.1. Precancerous Lesions

There is substantial evidence that HGSC can originate from either the ovarian surface epithelium (OSE) or the tubal epithelium (TE) of the uterine tube, also known as the fallopian tube in humans and oviduct in various animals [4,16,17,18,19,20]. Historically, HGSC was believed to originate from the ovarian inclusion cysts lined by OSE [19]. However, studies of familial HGSC cases discovered serous tubal intraepithelial carcinoma (STIC) lesions to be a common neoplastic precursor [4,21,22,23]. STICs exclusively form in the distal TE where they present as lesions lacking ciliation and expressing PAX8 [24]. These lesions are characterized by dysplastic epithelial cells with the loss of cell polarity, high proliferative index, and *TP53* missense or null mutations presented as increased p53 (also known as TP53 in humans and TRP53 in mice) accumulation or its total absence, respectively. Thus, clinical identification of STICs relies on hematoxylin and eosin staining, aberrant p53 immunostaining patterns, and increased number of Ki67^+^ neoplastic cells [25]. It is possible that STICs are preceded by the formation of “p53 signatures”, areas of a single layer of consecutive PAX8^+^ cells that contain aberrant p53 expression but lack cellular atypia and show a low proliferative index [22,24]. The extent of STIC and p53 signature contributions to sporadic non-familiar cases remains debatable. Thus, it is currently hypothesized that many cases originate in the TE, but both sites contribute to the HGSC pathogenesis [19,20].

Although the discovery of STICs in familial cases of HGSC facilitates research on earlier stages of HGSC, the contribution of precursor lesions to SEC is less clear. SEC is frequently associated with a putative precursor lesion known as serous endometrial intraepithelial carcinoma (SEIC). Such lesions are marked by atypical cells with aberrant p53 expression staining, consistent with *TP53* missense or null mutations [26,27]. SEICs are morphologically similar to overt SEC and are associated with metastatic disease development [27]. There is evidence that SEICs may be preceded by endometrial glandular dysplasia (EmGD) lesions [28]. EmGD lesions are described as single glands or glandular groups displaying intermediate focal cellular atypia and p53 signatures between the healthy endometrium and SEC [27,28]. Further research is required to evaluate the relationship between SEICs and EmGDs and their capacity to progress into SEC. 

Interestingly, the co-existence of SEC with STICs has been reported in 8–14% of cases [29,30,31]. Consistent with the possibility that STICs are a putative site of origin for some SECs, it has been reported that some cases of SEC may originate from TE secretory cells in a mouse model of HGSC [32]. In this model, HGSC and SEC form after *Myc* overexpression and *Trp53*-*R270H* mutation in *Ovgp1*^+^ TE secretory cells. The distinct origin of the majority of HGSC and SEC is also in agreement with differences in some recurrent genetic alterations, such as HGSC-specific *MDM2*, *CDK4*, *BRCA1*, and *BRCA2* alterations [33], in immunostaining markers, such as preferential expression of Wilms Tumor 1 in HGSC [14], and in the differential response of SEC to cisplatin, doxorubicin, and cyclophosphamide [34].

Taken together, both precursor lesions among the TE and endometrial epithelium (EE) share morphological similarities, p53 signatures, and markedly atypical cells. However, as discussed above, they also have notable differences. Further understanding of common and distinct properties of cancer-prone cells and their states is essential to the discovery of preventative and diagnostic markers of HGSC and SEC.

### 2.2. Ovarian Surface Epithelium

The OSE presents as a single layer of squamous epithelium capable of regeneration after ovulation [35]. The inactivation of *Trp53* (mouse homologue of human *TP53*) and accompanying genes, such as tumor suppressor *Rb1*, in 2D and 3D OSE cell cultures is sufficient to drive tumor formation when reimplanted within mice [16,36]. Similarly, intravital inactivation of *Trp53* and *Rb1* in OSE leads to the formation of HGSC [37]. Consistent with these observations, it has been reported that a gene signature indicative of OSE origin is associated with poor outcomes, and such tumors are both more resistant to chemotherapy and predisposed to a suboptimal debulking surgery as compared to TE-derived HGSC [18]. However, other studies claim that the mesenchymal subtype, typically associated with a poor prognosis, could be connected to diverse cell states present in TE cell lineages [17,38]. Inconsistency between reported gene signatures and the course of diseases in different studies suggests that the current stratifications of HGSC, according to their molecular profiles alone, are imprecise. 

**Figure 1:**
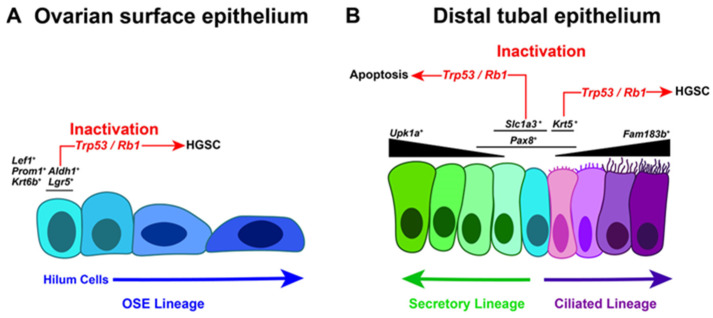
Cancer-prone cell states of the mouse ovarian surface epithelium (OSE) and distal tubal epithelium (DTE). (**A**) The differentiation trajectory of *Lgr5*^+^, ALDH1^+^ stem/progenitor cells for the (OSE) begins in the hilum and encompasses the whole OSE. Inactivation of *Trp53* and *Rb1* in *Lgr5*^+^ cells leads to high-grade serous carcinoma (HGSC). (**B**) The DTE has *Slc1a3*^+^ stem/progenitor cells that give rise to secretory (*Upk1a*^+^) and ciliated (*Fam183b*^+^) cell lineages. *Slc1a3*^+^ stem/progenitor cells do not transform and undergo apoptosis, while *Krt5*^+^ pre-ciliated cells do initiate HGSC after inactivation of *Trp53* and *Rb1*.

Further studies have identified OSE stem/progenitor cells as the main source of long-term OSE regeneration. Such cells are mainly located in the ovarian hilum region and are characterized by the expression of *Aldh1*^+^, *Lgr5*^+^, *Lef1*^+^, *Prom1*^+^, and *Krt6b*^+^ [39,40] (Figure 1). Compared to more differentiated OSE cells, stem/progenitor cells are more easily transformed by the inactivation of *Trp53* and *Rb1* and form HGSC [39,40,41]. These findings support the notion that OSE cells in a stem/progenitor state have a high propensity for malignant transformation, similar to cancer-prone stem cells of the hematopoietic system, intestinal tract, and skin [39,42,43]. Further evaluation of specific differentiation states of human OSE may shed more light on the potential contribution of OSE subpopulations to HGSC formation.

### 2.3. Tubal Epithelium

Identifying cells of origin within the TE is exacerbated by its cellular diversity as compared to the OSE, which is composed of a simple squamous epithelium. Immunophenotyping, lineage tracing, and single-cell RNA-sequencing (scRNA-seq) experiments suggest that regionally distinct cell lineages contribute to the distal and proximal uterine tube separately [44,45]. The distal TE is characterized by its abundance of ciliated cells, whereas the proximal TE consists predominantly of secretory cells. Other cells described within the distal TE are basal cells assumed to be intraepithelial lymphocytes and peg cells that either serve as progenitors or exhausted secretory cells [46].

Genetic cell lineage tracing experiments in mice identified that *Pax8*^+^ cells have the capacity to differentiate into ciliated cells in both the distal and proximal TE [47]. Consequently, *Pax8*^+^ progenitor cells have been shown to give rise to STICs and HGSC upon inactivation of *Trp53* with *Rb1* or with *Brca1*, *Brca2*, and *Pten* combinations within mouse models [48,49] (Figure 1). Oviductal glycoprotein 1 (OVGP1) is a marker of secretory cells within the TE, and inactivation of *Trp53*, *Brca1*, *Rb1*, and *Nf1* in *Ovgp1*-expressing cells also leads to STIC and HGSC within mouse models [50]. Recently, scRNA-seq analysis of mouse uterine tubes revealed that *Pax8* and *Ovgp1* were insufficiently specific markers of epithelial cell states in the distal TE [51]. *Slc1a3* was found to be a more distinct marker for stem/progenitors of the distal TE by scRNA-seq, organoid, and mouse lineage tracing studies [51]. Furthermore, inactivation of *Trp53* alone or together with *Rb1* in *Slc1a3*^+^ stem/progenitors led to apoptosis rather than HGSC, whereas *Krt5*^+^ pre-ciliated cells expressing *Prom1* and *Trp73* (mouse homologue of *TP73*) did lead to STIC formation and HGSC after inactivation [51]. *Prom1*^+^ cells have also been reported by other lineage-tracing studies in mice to be capable of rapid transition into ciliated cells [52]. 

The characterization of human TE cell states remains incomplete due to a failure to reach a consensus on the cell states present within the TE. Human scRNA-seq analyses have met inconclusive results, with discrepancies over lineage dynamics and transitional cell states [53,54,55,56]. Specifically, *RUNX3*^+^ cells were described as transitional pre-ciliated cells within one study, while another study identified *RUNX3*^+^ cells as basal cells [54,55]. Further debate exists among progenitor and lineage dynamics, where some claim that *KRT17*^+^ secretory cells are progenitors, while others believe there are two distinct lineages contributing to ciliated and secretory cells separately [53,54,55]. Among the scRNA-seq analyses, mechanisms involved with ciliogenesis were revealed to share expression with ovarian cancer risk genes, suggesting that ciliated cells are implicated in the origin of HGSC in humans as well [56]. Consistent with mouse data, there is further evidence that the ciliated cell lineage is involved with HGSC as ciliogenesis-related *TP73* is upregulated in epithelial ovarian cancers [57]. However, more research is needed to identify cellular origins of HGSC in the human TE. Mouse models point to a pre-ciliated cell state as a cell of origin, and a conclusive cell-state hierarchy within humans can enable direct comparison of mouse studies to the discovery of a cell of origin in humans.

### 2.4. Endometrial Epithelium

**Figure 2:**
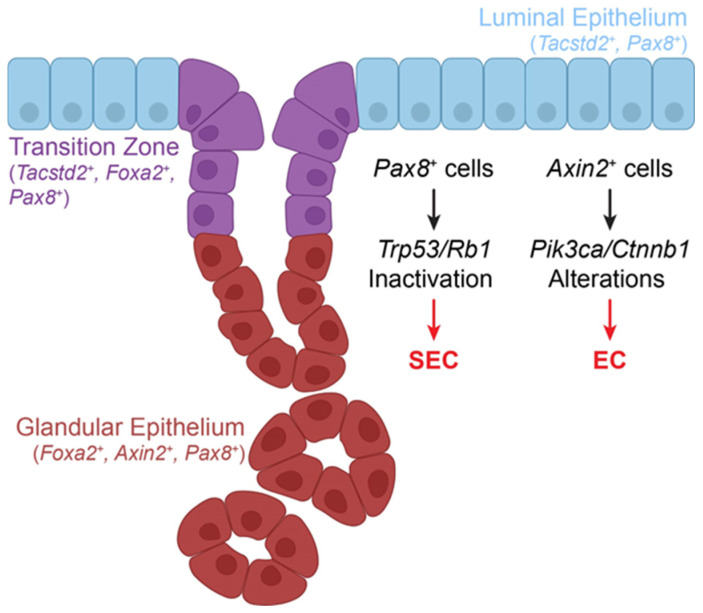
Cell composition of the mouse endometrial epithelium. Putative cancer-prone sites include luminal epithelium (LE; blue, columnar cells), transition zone (purple cells), and glandular epithelium (GE; red, cuboidal cells). The inactivation of *Trp53* and *Rb1* in *Pax8*^+^ cells results in serous endometrial carcinoma (SEC). *Pax8* is expressed in both the LE and GE. It plays a critical role in the development of Müllerian duct-derived tissues, such as the uterus and uterine tube. The glandular epithelium contains *Axin2*^+^ cells, which are needed for proper glandular development. When an oncogenic form of *Pik3ca* and overexpression of *Ctnnb1* are introduced to these cells, endometrial carcinoma (EC) of an unspecified subtype develops.

The endometrium contains two distinct epithelial layers: the glandular epithelium (GE) and the luminal epithelium (LE; surface epithelium in humans) (Figure 2). Mouse LE and GE are marked by expression of TROP2 (encoded by *Tacstd2*) or FOXA2, respectively [48]. Both layers contain ciliated and secretory cells much like the TE [58]. Putative stem/progenitor markers of the endometrium include *Pax8*, *Axin2*, and *Lgr5* [48,59,60,61]. 

Concurrent inactivation of *Trp53* and *Rb1* in *Pax8*^+^ cells (Pax8-model) results in SEC, in parallel with HGSC formation [48,51], which highlights the similarities in SEC and HGSC pathogenesis. In the Pax8-model, over 80% of mice developed SEC, with progression to an invasive stage within 180 days, and 45% of mice developed TE neoplastic lesions by 300 days after gene inactivation. As *Pax8*^+^ cells are present in both GE and LE, further identification of cancer-prone subpopulations is required [48]. To capture the transcriptomic differences between the healthy, pre-dysplastic and dysplastic stages of SEC, the Pax8-model was further explored using scRNA-seq [62]. Immature-like cells that were frequently positive for the LE-specific marker *Tacstd2* increased in frequency as SEC progressed. At the protein level, TROP2 expression increased in the GE. Interestingly, the expansion of progenitor populations and TROP2 expression in the GE were identified prior to dysplastic lesions forming in the endometrium [62]. This suggests that either malignant cells preferentially adopt a LE-like phenotype or that the LE cells are predisposed to SEC malignant transformation. 

Conversely, a population within the GE is susceptible to malignant transformation. Cells expressing *Axin2*, a Wnt signaling response gene, are reportedly responsible for glandular development and regeneration [59]. These cells can also produce organoids that express markers of ciliation, proliferation, and the GE, which could be maintained for multiple passages [59]. Endometrial carcinoma (EC) develops in the presence of mutant *Pik3ca* alongside activation of the Wnt pathway through *Ctnnb1* expression in *Axin2*^+^ cells. Unfortunately, the subtype of EC was not reported in this mouse model [59]. Nevertheless, based on the stem-like properties of *Axin2*^+^ cells, these data suggest that the stem/progenitor states within the endometrium are particularly susceptible to malignant transformation and implicate the Wnt signaling pathway in EC pathogenesis. 

As previously stated, *Lgr5*^+^ stem/progenitor cells in the mouse OSE hilum can initiate HGSC. Research on mice and humans suggests that LGR5^+^ cells could serve as putative stem/progenitor cells in the endometrial epithelium [58,60]. LGR5^+^ cells can efficiently form endometrial organoids, further supporting their stem-like qualities [61]. However, no endometrial neoplastic lesions were observed by 600 days after inactivation of *Trp53* and *Rb1* [41], although it remains possible that combinations of other genetic alterations would lead to endometrial carcinoma. 

Cancer-prone pre-ciliated cell states described in the TE could also exist as a susceptible cell state in the endometrium. In humans, the majority of ciliated cells are believed to reside in the surface epithelium but can also be found in the GE [58,63]. The abundance of ciliated endometrial cells substantially increases with age [64]. Endometrial organoids have demonstrated their ability to recapitulate ciliation ex vivo and have improved a molecular understanding of endometrial ciliation [65]. Organoids require a high Wnt-to-Notch signaling ratio and estrogen stimulation to drive ciliation [58,65]. While exogenous estrogen exposure is not a risk factor for SEC [26], disruption of Notch and Wnt signaling is associated with poor prognosis of EC [66,67]. Cells with high Wnt-to-Notch signaling ratios may lack the estrogen levels needed for cells to progress past a pre-ciliated state, which may increase transformation potential. However, Wnt signaling alterations are more common in endometrioid endometrial carcinomas than serous [68]. Further research informed by HGSC pathogenesis could clarify the contribution of ciliated cell states in SEC.

In contrast, Notch signaling drives secretory differentiation within the endometrium. When considering typical Notch signaling disruption in SEC, poor prognosis is frequently associated with overexpression of NOTCH1 and/or NOTCH2 [66,69], lending credence to secretory cell states being susceptible to SEC initiation. Further studies are needed to characterize the potential contribution of secretory endometrial cells to endometrial carcinoma. 

A definitive characterization of cell states among the LE and GE and specific ciliated or secretory lineages in the human and mouse endometrium is yet to be completed. The work in this area will facilitate identification and characterization of cancer-prone cell states. Fortunately, pathogenesis of SEC and HGSC is similar but not identical. Thus, comparative evaluation of TE and EE may facilitate the identification of common and divergent mechanisms of carcinogenesis in both epithelia.

## 3. Genetic Drivers

Genomic sequencing of HGSC and SEC tumor samples has been instrumental in identifying genetic drivers of theses cancers [33,68]. Genetic alterations induce transformation and malignant characteristics such as a loss of cell cycle control (RB1 pathways), increased cellular proliferation (RAS and PI3K pathways), and DNA repair deficiencies (HRD pathways). 

**Figure 3:**
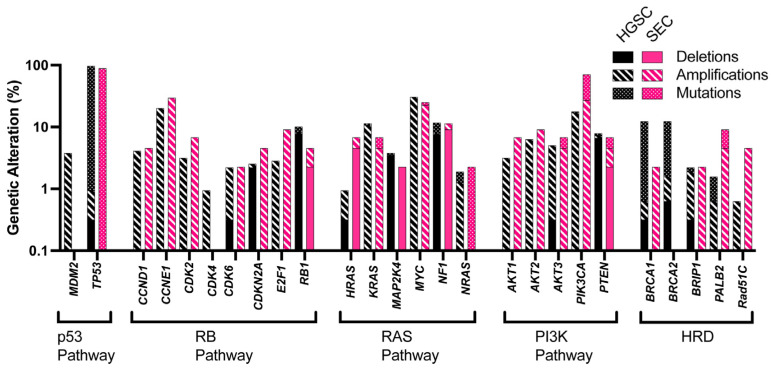
Comparison of genetically altered gene frequency between high-grade serous carcinoma (HGSC) and serous endometrial carcinoma (SEC). Genetic alterations are defined as gene mutations, amplifications, and deep deletion events. A total of 316 HGSC [33] samples and 44 SEC [68] samples were analyzed (TCGA datasets extracted via the cBioPortal interface) and plotted on a logarithmic scale. Note lack of *MDM2*, *CDK4*, *BRCA1*, and *BRCA2* alterations in SEC.

The tumor suppressor gene *TP53* is mutated in most HGSCs and SECs (Figure 3). p53 has a critical role in cell cycle progression, apoptosis, genomic stability, and senescence. Supporting the critical role of p53 alterations in the early stages of carcinogenesis, almost all STIC and SEIC lesions carry *TP53* mutations [3,27,33,68]. Other mutations in p53 pathway, such as the amplification of *MDM2* (a negative regulator of p53) [70] or dysregulation of *mir-34* (a microRNA regulated by p53), also promote ovarian, prostate, and breast cancers [71,72,73]. However, previous studies have demonstrated that *Trp53* loss in mice and organoid models is necessary but insufficient for initiating HGSC [16,36,37,40,70]. 

The RB1 pathway is also commonly dysfunctional in HGSC and SEC [74,75,76] (Figure 3). This pathway includes the tumor suppressor genes *RB1* and *CDKN2A* and the oncogenes *CCND1*, *CCNE1*, *CDK2*, *CDK4*, *CDK6*, and *E2F1*. Together they control cell cycle progression, chromatin remodeling, and senescence [77,78]. Genetically altered *RB1* is observed in 10.13% of HGSC [33] and 4.55% of SEC [68,79], respectively. *CDKN2A* is also genetically altered at a frequency of 2.53% in HGSC and 4.55% in SEC. Overall, the RB1 pathway gene alterations are observed in 35.76% of HGSC and 45.45% of SEC. Concurrent loss of *Trp53* and RB1 pathway-related genes in genetically modified mice result in HGSC [16,39] and SEC [48,62]. These data suggest that loss of *TP53* and alterations in the RB1-related pathway are essential for both HGSC and SEC.

In addition to alterations in *Trp53* and RB1 pathway-related genes, HGSC and SEC contain a number of other concurrent alterations that may further facilitate carcinogenesis [40]. By using CRISPR-Cas9 mutagenesis in TE and OSE, the combinations of key gene drivers for HGSC development have been identified [36,40,80].

Disruption of the RTK–RAS–MEK pathway allows transformed cells to undergo rapid and uncontrolled proliferation and is common across many cancers, including HGSC and SEC (Figure 3). *Nf1* mutations have been shown to drive HGSC and other cancers [36,79,81]. However, *KRAS* mutations have been associated with low-grade serous ovarian carcinomas [82,83]. Both HGSC and SEC have similar rates of genetic perturbations of RAS pathway-related genes [33]. MYC, a downstream factor of this pathway, is often overexpressed in HGSC and SEC. Overexpression of MYC in combination with other mutations has been validated in mouse models as a driver of HGSC [16]. Loss of *Nf1* in *Trp53*-deficient TE cells also results in an HGSC-like phenotype [80,84]. The impact of MYC and NF1 alterations on SEC formation in mice remains to be clarified. 

Both HGSC and SEC also have alterations in the PI3K pathway regulating cell proliferation and survival (Figure 3). However, their contributions significantly differ for some genes. For example, amplified *PIK3CA* is detected in 17.08% of HGSC and 27.27% of SEC. *PTEN* mutations are more common in endometrioid types of ovarian and endometrial carcinomas but are also observed in advanced stages of HGSC [33] and SEC [85]. *PTEN* deletion is observed in 6.65% of HGSC and 2.27% of SEC. *PTEN* loss also causes PARP inhibitor sensitivity reversion [36]. PTEN mutations have been validated as a driver of transformation in mouse models. However, secondary mutations are necessary for the most efficient transformation in such models [80]. 

Mutations causing homologous recombination deficiency (HRD) occur in over 50% of epithelial ovarian and 22% of endometrial carcinomas manifesting HRD, respectively [76]. HRD supports the accumulation of mutations aiding cell survival in an ever-changing tumor microenvironment and in response to common therapeutics. Pan cancer studies have identified *Brca1*, *Brca2*, *Brip1*, *Rad51C*, and *Palb2* as the most commonly mutated genes related to HRD [86,87,88] (Figure 3). Additionally, patients with *BRCA1* mutations are more likely to be long-term survivors and have a higher mutational burden compared to patients lacking *BRCA1* mutations [76,89]. Surprisingly, loss of *BRCA1/2* in a *Trp53*-deficient background in mouse OSE and TE cells was insufficient to transform both cell types and did not have tumorigenic potential, suggesting that further mutations are necessary for tumorigenic potential [36,40,80]. There is some evidence that *BRCA1/BRCA2* mutations play a role in endometrial cancers [90,91]. Patients subjected to salpingo-oophorectomy due to germline *Brca1/Brca2* mutations have an increased risk for the development of SEC [92]. However, the causative role of *Brca1/Brca2* mutations in SEC remains to be clarified.

In summary, a loss of cell cycle control, increased cellular proliferation, and DNA repair deficiencies aid in the initiation and progression of HGSC and SEC. Various mutational combinations yield similarities and differences among the requirements for a transformation within TE and OSE. Many of the genes identified in HGSC have been well studied and characterized. However, their combinations driving SEC remain to be elucidated. Further work is necessary to determine whether LE and GE in the endometrium share the same combinations of drivers as TE and/or OSE.

## 4. Future Directions

Discovering cancer-prone cell states is limited by the model systems necessary to approach this area of research. Identification of new cell state-specific promoters and cell fate tracing systems should facilitate advancements in studying what cell states can initiate the disease [93]. Another important experimental aspect is the development of animal models that better accommodate the differences between human and mouse reproductive systems. Such improvements may involve using aged and oophorectomized mice to better model the postmenopausal age of most HGSC and SEC patients. Another promising approach is to use menstruating spiny mice, which better mimic the native mechanisms of cell turnover in the human reproductive tract [94]. 

Organoids serve as ex vivo models that better recapitulate the architecture and cellular diversity of native human tissue than 2D cultures. However, organoid models may lack the complexity necessary to study additional factors contributing to carcinogenesis. Assembloid models incorporate multiple cell types to more accurately model human tissue, and these models could provide sufficient complexity to evaluate how additional cell types interact with transformed cell states [93,95]. 

Additionally, rapidly advancing research efforts in computational approaches are at the forefront of tissue characterization and biomarker identification [96,97,98]. The increased resolution, approaching sub-micrometer, within spatial transcriptomic platforms can allow for direct comparison among the epithelial layers of the reproductive tract [99,100]. Combinatorial approaches with genomic, transcriptomic, and epigenomic data applied to a subcellular spatial context at early stages of disease development will be pivotal for the discovery of diagnostic and therapeutic targets. Further development of new computational approaches to compare data among mouse and human tissues should facilitate direct translation of findings from mouse models to humans, while leveraging the flexibility of early disease detection only possible in model systems. Current examples of this include analysis tools such as the self-assembling manifold (SAM) algorithm, which uses BLAST to map homology between datasets of distinct species to identify shared expression programs [101]. New methods combining lineage tracing and single-cell and spatial RNA-sequencing data could provide insight into cell lineage trajectories, susceptible cell states, and tumor evolution by actively tracking differentiation patterns and accumulated mutations [102]. Higher resolution of spatial transcriptomics applied to STIC/SEIC lesions could inform the cell states implicated in both the formation and progression of early HGSC and SEC. The identification of new markers and epigenetic modifications associated with developing tumors could be leveraged with early detection technologies that analyze methylation patterns of circulating DNA [103], such as the GRAIL’s Galleri test [104]. The continued advancement in biomedical research technology could allow for the identification of early markers within cancer-prone cell states necessary for disease prevention and early intervention of serous carcinomas. 

Unbiased CRISPR combinatorial screens have revealed genetic perturbations that are critical for biological processes [105]. Much effort has been made to understand how combinations of genes result in the transformation of healthy cells into malignant cells in HGSC [40]. Such screening approaches have yet to be performed in SEC. These studies will show whether the same combinations of genetic drivers in HGSC are also shared in SEC. Additionally, they would determine whether unique sets of combinations specific to SEC suggest differences in cell of origin. This may explain why the development of SEC in women with *BRCA1/2* mutations is only seen following salpingectomy. Many combinatorial screening approaches have been performed in mouse models; therefore, the use of human cells for similar screens may better reflect the minimum transformation requirements of these cell types.

Transformation of healthy cells is driven not only by loss of gene functions but also by amplification/and gene overexpression or gain of function mutations events. Using CRISPR-based activation (CRISPRa) [106], gene overexpression can be studied to identify the combinations of amplified genes required for transformation. Additionally, screens to test the minimum transformation requirement have only been performed; however, utilizing such strategies to identify the most chemotherapy-resistant combinations may help to improve patient outcomes. Development of new approaches combining genetic perturbation approaches with computational analysis [97,107] will further facilitate our understanding of tumor suppressor and oncogene networks, thereby improving our understanding of both serous carcinoma biology and patient survivability.

## 5. Conclusions

Taken together, it is increasingly clear that not only cell type but also state of cell differentiation in conjunction with specific genetic alterations define resulting cancer types, their biological behavior, and their response to treatment. Further work in this direction should greatly benefit the progress towards curing aggressive serous carcinomas of the female reproductive system.
